# Large Cell Neuroendocrine Prostate Cancer: Large Is Not Small

**DOI:** 10.1093/oncolo/oyad344

**Published:** 2024-01-11

**Authors:** Anthony V Serritella, Himisha Beltran, Tamara L Lotan, David J VanderWeele, Fatima Karzai, Ravi A Madan, Maha Hussain

**Affiliations:** Robert H. Lurie Comprehensive Cancer Center; Northwestern University Feinberg School of Medicine, Chicago, IL, USA; Dana-Farber Cancer Institute, Harvard Medical School, Boston, MA, USA; Department of Pathology, Department of Urology, Department of Oncology, Johns Hopkins University School of Medicine, Baltimore, MD, USA; Robert H. Lurie Comprehensive Cancer Center; Northwestern University Feinberg School of Medicine, Chicago, IL, USA; Genitourinary Malignancies Branch Center for Cancer Research, National Cancer Institute, National Institutes of Health, Bethesda, MD, USA; Genitourinary Malignancies Branch Center for Cancer Research, National Cancer Institute, National Institutes of Health, Bethesda, MD, USA; Robert H. Lurie Comprehensive Cancer Center; Northwestern University Feinberg School of Medicine, Chicago, IL, USA

## Abstract

Neuroendocrine prostate cancer is complex, comprising a wide spectrum of phenotypes, which has led to very different entities being inappropriately lumped together or assumed to behave like more common entities. Elucidating subtle differences is critical for treatment decision making, as this commentary describes.

Approximately 1% of patients with prostate cancer (PC) have pure neuroendocrine (NE) histology at diagnosis,^1^ and 15%-20% of later-stage patients may develop PC with NE features.^2,3^ The term “neuroendocrine PC” (NEPC) has been used to encompass any PC with NE features based on morphology and/or immunohistochemical staining (IHC).^4^ Most oncologists are aware of NEPC’s general characteristics, including rapid and often visceral metastatic progression in the setting of low or non-rising prostate-specific antigen (PSA), and NE marker expression (eg, chromogranin, synaptophysin, and/or insulinoma-associated- protein-1 [INSM-1]).^5^

However, NEPC is complex, comprising a wide spectrum of phenotypes. This has led very different entities to be inappropriately lumped together or simply assumed to behave like the most common NEPC entities. Elucidating subtle differences between various NEPCs is critical for treatment decision-making.^6-10^

This commentary was triggered by a 62-year-old gentleman who presented with a PSA of 134.71 ng/mL and imaging demonstrating pulmonary nodules, multiple liver masses, and diffuse bone metastases. Prostate and liver biopsies were read as “high-grade prostatic adenocarcinoma with extensive NE differentiation.” Tumor tissue stained positive for synaptophysin, chromogranin, and PC luminal lineage marker NKX3-1. He was treated by his oncologist with carboplatin, etoposide, androgen deprivation therapy (ADT), and enzalutamide. After 4 months his PSA started rising and a second review of the original tissue at our institution revealed a diagnosis of large-cell NEPC (LCPC). His disease progressed on docetaxel + carboplatin and subsequently cabazitaxel, as well as one dose of lutetium-177 PSMA-directed therapy. He expired due to fulminant liver failure related to progression. His survival was 16.5 months from diagnosis.

## Neuroendocrine Differentiation Does Not Imply Small Cell or Large Cell Carcinoma

Critical to managing NEPC is determining whether an actual NE *disease variant requiring different treatment* is present. NE differentiation occurs in benign prostate glands—scattered NE cells producing peptide growth hormones are often present.^8,11^ In conventional prostatic adenocarcinoma, foci of NE differentiation in an otherwise morphological adenocarcinoma does not necessarily imply adverse prognosis.^12,13^ NE features are assumed by many to automatically indicate aggressive features which is not always the case. However, since NE staining is typically not requested without concern for aggressive disease, oncologists may inappropriately conclude all incidences of NE features are an ominous finding.

In contrast, the NE *variant* of PC (NEPC) is much rarer, associated with a poorer prognosis^1,2,14-16^ and the incidence increases after ADT. Treatment-related NEPC can occur as early as 24 months after starting ADT.^1,6,17^ NEPC is often associated with aggressive molecular features like *TP53* and *RB1* loss, high Ki67, epigenetic alterations, and growth despite androgen receptor (AR) inhibition.^18^

The presence of NE features should not automatically lead to NEPC type of therapy, ie, small-cell cancer therapy. Establishing whether there is a *pathologic* NE variant, and interpreting this finding in the appropriate clinical context, are necessary when deciding whether a different therapeutic approach should be taken.

Three major poorly differentiated NE subtypes have been described in PC, though this current pathologic classification is subject to great variability, even amongst expert pathologists. Small-cell NE carcinoma (SCPC), LCPC, and adenocarcinoma with NE features with either mixed-morphology or overlapping features (ie, amphicrine) are terms that are often used clinically. In addition to tumor morphology, IHC staining for classical PC and NE markers is also typically performed.

SCPC can be challenging to accurately diagnose as its clinical/histological features have only gained traction in recent years.^19^ While de novo SCPC can occur, SCPC typically arises after periods of ADT. Given similarities with small-cell lung cancer (SCLC), SCPC is treated with platinum and etoposide with a response rate as high as 50%-60%.^4^ In recent years, adding immunotherapy further improved outcomes.^20^ Unlike SCPC, adenocarcinoma with NE features often continues to respond to standard prostate cancer therapies.

LCPC is a distinct, extremely rare NEPC with only ~20 cases described in the literature.^7,9,10,21^ LCPC is very aggressive, with a median survival of ~15 months.^7,22^ Pathologists and oncologists often do not recognize LCPC as a distinct entity, as the prognostic and therapeutic implications of the diagnosis are unclear. Importantly, the presence of NE features must not trigger physicians to simply treat LCPC like SCPC.

LCPC is generally under-recognized and underreported for several reasons.^7-10,21,23^ First, due to LCPC’s rarity, pathologists will often simply report LCPC tumors as generically “poorly differentiated high-grade PC” without noting specific large-cell characteristics. Secondly, LCPC is often found incidentally, in high disease burdens requiring palliative procedures where tissue may otherwise not normally be assessed.^7,21^ Finally, diagnostic criteria for LCPC are more strict^8^ making LCPC’s *precise* diagnosis difficult to make. LCPC’s incidence is challenging to approximate but likely more prevalent than currently reported.

## Treatment Recommendations for LCPC

We recommend that treatment decisions for LCPC should be based on which of three LCPC disease forms are present^7^: (1) de novo LCPC *without* admixed adenocarcinoma, (2) de novo LCPC *with* admixed adenocarcinoma, and (3) LCPC arising after ADT/AR-directed therapy.

Physicians should not prematurely abandon traditional PC treatments simply because large-cell differentiation is present.^3^ It is not advisable to prematurely forgo valuable AR-directed therapies in favor of small-cell chemotherapy in all patients. The expression of AR and AR signaling markers may help guide this decision.

De novo LCPCs, without any ADT history, are exceptionally rarer than post-ADT forms.^7,24^ Pure de novo LCPC, not associated with adenocarcinoma, often has a larger disease burden and worse prognosis. In general, NEPC typically loses AR (or related gene) expression, secrete little-to-no PSA and may require chemotherapy.^24^

By contrast, admixed LCPC typically retains AR dependence and secretes PSA. As a result, localized disease may be caught before systemic spread, potentially allowing for curative therapy. With regards to metastatic disease, 3 reported cases of de novo LCPC treated with ADT had responses in the range of 1-2 years.^24^

LCPC retains some degree of androgen dependence as it can present with a high PSA that decreases with ADT. Two patients at our institution with de novo LCPC had an OS ~5 years post-treatment with ADT + conventional therapies (enzalutamide, abiraterone, docetaxel, and cabazitaxel).^22^ Thus a positive response *may* potentially be from both AR-directed therapy and chemotherapy.

Though data are very limited, many LCPC-reported cases occur after longstanding ADT, sometimes 4-5 years after the original PC diagnosis.^7,8,10^ Post-ADT LCPC, like post-ADT SCPC or other high-grade NEPCs, may respond better to chemotherapy since they may demonstrate low or absent AR, NKX3.1, and PSA expression and are less likely to respond to AR-targeted therapy.^7,8^

LCPCs may respond to platinum agents or taxanes^25^ potentially due to tumor suppressor gene loss or DNA repair deficiencies.^4^ Cabazitaxel combined with platinum shows a response in many NEPCs, including LCPC^26^ likely due to cabazitaxel activity in both CRPC and mixed-tumor histologies.^4^ Retrospective data suggest checkpoint inhibitors should be explored further.^27^

## Guide for Histologic Diagnosis

Tables 1 and 2 summarize some of the major histologic differences between SCPC and LCPC as well as de novo vs. post-ADT LCPC.

**Table 1.** Histologic guide for differentiating LCPC and SCPC.

Feature	LCPC	SCPC
Cellular morphology	Large cells with abundant cytoplasm Large nests, sheets and cords with peripheral palisading Geographic necrosis	Small cells with a high nuclear: cytoplasmic ratio Significant crush artifact Apoptosis and necrosis
Tumor nuclei	Coarse, clumpy chromatin	Nuclear molding
Nucleoli	Prominent	Absent
Mitotic rate	High (Ki-67 >60%)	High (Ki-67 >60%)
Requirements for diagnosis	Specific morphologic features and strong IHC for ≥1 NE marker	Wider spectrum of suggestive cytologic features without specific IHC requirements
IHC	Typically negative staining or only focal positivity for luminal markers (PSA, PSAP)	Typically negative staining or only focal positivity for luminal markers (PSA, PSAP)

**Table 2.** Immunohistochemical differences between LCPC forms.

IHC	Post-ADT LCPC	De Novo LCPC
Synaptophysin IHC	++	+
Chromogranin-A	++	+
CD56	+	++

Plus signs (+) indicate relative levels of expression by the indicated assay.

SCPC typically has relatively small cells (<3 lymphocyte diameters), a high nuclear-to-cytoplasm ratio, and salt-and-pepper chromatin with small (or absent) nucleoli.^28^ Nuclear molding is frequently present, with tumors in large sheets, trabeculae, or acinar growth patterns. Tumors tend to be mitotically active, with a high proliferation index.

In contrast, LCPC cells are larger and have abundant cytoplasm, coarse/clumpy nuclear chromatin and prominent nucleoli.^6-8,10^ LCPC cells exhibit NE architecture and markers with cells arranged in large nests, sheets, or cords with peripheral palisading. While LCPC cells demonstrate brisk mitotic activity (often with geographic necrosis),^9^ mitotic activity may be lower than in SCPC.^23^

The *2013 Prostate Cancer Foundation Working Group on Neuroendocrine Differentiation* developed diagnostic criteria for LCPC: cells were required to express at least one NE marker by IHC and show *specific* morphologic characteristics (eg, large nests with peripheral palisading).^8^ This makes LCPC’s diagnosis rare by design, to sharply differentiate it from more generic poorly-differentiated adenocarcinoma with NE features.

A 2010 publication by Aparicio et al^29^ hypothesized that LCPC may represent a cytologically intermediate phase in the morphologic evolution from adenocarcinoma to SCPC. Whether LCPC truly represents a distinct entity, versus a mere transition, is unclear.

## Recommendation for Immunohistochemical Diagnosis

Some key IHC differences separate de novo and post-ADT NEPCs (Tables 1 and 2). Post-ADT LCPC can have a lower expression of synaptophysin/chromogranin and a higher CD56 expression than de novo cases.^7^ Lower/absent expression of PSA, PSAP, and AR is common, compared with de novo LCPC.

Typical IHC panels include chromogranin, synaptophysin, CD56, and the newer marker, INSM-1.^23^ In lung cancer, synaptophysin, chromogranin, and CD56 have lower sensitivity and specificity for small-cell or large-cell carcinoma.^30-32^ Comparing SCLC to large-cell lung cancer, synaptophysin (41%-75% vs. 58%-85%), chromogranin (23%-58% vs. 42%-69%), and CD56 (72%-99% vs. 72%-94%) had relatively similar expression.

In PC, chromogranin or synaptophysin staining is neither sensitive nor specific for NEPC. In SCPC, synaptophysin expression is seen in ~85%, but chromogranin expression occurs in ~55%.^23^

INSM-1 more sensitively detects SCPC than LCPC (93.9% vs. 62.5%, *P* = .015), with higher specificity (97.4%) for detecting *any* genitourinary NE carcinomas.^33^ In SCPC, INSM-1 is upregulated in as many as 77%-90% of cases, with 95% specificity.^34^ By contrast, non-NE prostatic tissues generally lack INSM-1 expression.

A large series of a variety of genitourinary malignancies reported INSM-1 expression of 21% in LC tumors, lower than typically seen in SCPC.^33^ This trend was also found in lung and other malignancies. Therefore INSM-1 staining *might* differentiate SCPC from LCPC, but there is still limited data.

## LCPC Molecular Features

Given LCPC’s rarity, its molecular characterization is still investigational. We recently completed a retrospective study of 6 patients with de novo LCPC at our institution.^22^ Patients were all microsatellite-stable, had *TP53* mutations, *PTEN* loss, and *Rb1* loss. Fifty percent of our cases coexisted with a grade 5 adenocarcinoma and were not entirely NE in composition. These alterations are similar to those found in SCPC. Future work is necessary to understand if there are molecular markers that differentiate LCPC from other NEPCs to potentially provide new therapeutic targets.

## Conclusions and Recommendations for Future Investigation

1. *Increase awareness of LCPC:* Improved awareness could guide oncologists to better understand the spectrum of NEPC and not reflexively treat all patients like SCPC.^21^ Importantly, oncologists should not forgo life-prolonging AR-directed therapy simply because LCPC is identified, especially if AR and AR signaling is present.
2. *Need better pathologic criteria:* NE-tumor diagnosis rests on morphological, functional, and IHC criteria, subject to interobserver variation.^21^ Pathology reports often incorrectly lump together NEPCs, masking the distinctive presence of different subtypes.^8,13,35,36^ Improved criteria, including molecular markers, could potentially help distinguish clinically relevant subtypes of NEPCs. We recommend not focusing on any one feature in isolation, instead integrating morphology, IHC markers, and the cell-cycle axes to aid in diagnosis, and in interpreting these features in the clinical context of the patient (eg, PSA and aggressiveness of the disease).
3. *Accounting for NE admixture:* Percentage and grade of the adenocarcinoma component should be provided on pathology reports.^6^
4. *Need for better molecular biomarkers*: Genomic alterations in *TP53*, *RB-1*, *PTEN*, and *TMPRSS2-ERG* fusions are often found in aggressive traditional PC and other NEPCs.^6^ Determining LCPC-specific genetic/epigenetic evolution may help predict a patient’s future trajectory.^3^ New biomarkers might identify large-cell NE differentiation before advanced-disease develops. While most NEPC features are typically not detected early, there may be early transcriptomic changes associated with AR independence.^37,38^ Most targetable alterations are acquired after therapy. Cell-free DNA (cfDNA) may detect NEPC-associated DNA methylation changes over time.^39-41^ Repeat biopsies on progression should be considered.
5. *Cell of origin:* Whether disease originates after long-term hormonal pressure versus de novo disease may have implications on clinical decisions. While most NEPCs typically develop after long periods of treatment, this does not explain de novo disease or why certain forms retain AR susceptibility. There have not been substantial investigations into cells of origin or the molecular genesis for less common pure NE malignancies. Improved understanding of cellular origins may inform treatment.
